# Airborne Disinfection by Dry Fogging Efficiently Inactivates Severe Acute Respiratory Syndrome Coronavirus 2 (SARS-CoV-2), Mycobacteria, and Bacterial Spores and Shows Limitations of Commercial Spore Carriers

**DOI:** 10.1128/AEM.02019-20

**Published:** 2021-01-15

**Authors:** Jan Schinköthe, Hendrik A. Scheinemann, Sandra Diederich, Holger Freese, Michael Eschbaumer, Jens P. Teifke, Sven Reiche

**Affiliations:** a Department of Experimental Animal Facilities and Biorisk Management, Friedrich-Loeffler-Institut, Greifswald-Insel Riems, Germany; b Institute of Novel and Emerging Infectious Diseases, Friedrich-Loeffler-Institut, Greifswald-Insel Riems, Germany; c Institute of Diagnostic Virology, Friedrich-Loeffler-Institut, Greifswald-Insel Riems, Germany; University of Bayreuth

**Keywords:** airborne disinfection, dry fog, room disinfection, peroxyacetic acid, severe acute respiratory syndrome coronavirus 2 (SARS-CoV-2), Indiana vesiculovirus (VSIV), murine norovirus (MNV), *Mycobacterium senegalense*, *Geobacillus stearothermophilus*, *Bacillus subtilis*

## Abstract

Airborne disinfection of high-containment facilities before maintenance or between animal studies is crucial. Commercial spore carriers (CSC) coated with 10^6^ spores of Geobacillus stearothermophilus are often used to assess the efficacy of disinfection. We used quantitative carrier testing (QCT) procedures to compare the sensitivity of CSC with that of surrogates for nonenveloped and enveloped viruses, including severe acute respiratory syndrome coronavirus 2 (SARS-CoV-2), mycobacteria, and spores, to an aerosolized mixture of peroxyacetic acid and hydrogen peroxide (aPAA-HP). We then used the QCT methodology to determine relevant process parameters to develop and validate effective disinfection protocols (≥4-log_10_ reduction) in various large and complex facilities. Our results demonstrate that aPAA-HP is a highly efficient procedure for airborne room disinfection. Relevant process parameters such as temperature and relative humidity can be wirelessly monitored. Furthermore, we found striking differences in inactivation efficacies against some of the tested microorganisms. Overall, we conclude that dry fogging a mixture of aPAA-HP is highly effective against a broad range of microorganisms as well as material compatible with relevant concentrations. Furthermore, CSC are artificial bioindicators with lower resistance and thus should not be used for validating airborne disinfection when microorganisms other than viruses have to be inactivated.

**IMPORTANCE** Airborne disinfection is not only of crucial importance for the safe operation of laboratories and animal rooms where infectious agents are handled but also can be used in public health emergencies such as the current severe acute respiratory syndrome coronavirus 2 (SARS-CoV-2) pandemic. We show that dry fogging an aerosolized mixture of peroxyacetic acid and hydrogen peroxide (aPAA-HP) is highly microbicidal, efficient, fast, robust, environmentally neutral, and a suitable airborne disinfection method. In addition, the low concentration of dispersed disinfectant, particularly for enveloped viral pathogens such as SARS-CoV-2, entails high material compatibility. For these reasons and due to the relative simplicity of the procedure, it is an ideal disinfection method for hospital wards, ambulances, public conveyances, and indoor community areas. Thus, we conclude that this method is an excellent choice for control of the current SARS-CoV-2 pandemic.

## Abstract (precis)

Airborne disinfection is not only of crucial importance for the safe operation of laboratories and animal rooms where infectious agents are handled but also can be used in public health emergencies such as the current severe acute respiratory syndrome coronavirus 2 (SARS-CoV-2) pandemic. We show that dry fogging an aerosolized mixture of peroxyacetic acid and hydrogen peroxide (aPAA-HP) is highly microbicidal, efficient, fast, robust, environmentally neutral, and a suitable airborne disinfection method.

## INTRODUCTION

The disinfection of laboratories and animal rooms in high-containment environments is complex, and effective procedures are required to render an area biologically safe, e.g., between animal studies or for maintenance access. In most settings, after wet chemical cleaning and disinfection of easily accessible surfaces, airborne disinfection with gaseous formaldehyde (1, 2) or vaporized hydrogen peroxide is conducted to reach otherwise inaccessible surfaces (3–5). Besides the effectiveness, the corrosiveness of these disinfection methods must also be assessed. Here, we compared the efficacy of an aerosolized mixture of peroxyacetic acid and hydrogen peroxide (aPAA-HP) against a wide range of microorganisms under different conditions. The dispersion of aPAA-HP in ultrafine particles has recently been described as very effective (6, 7). In general, liquid PAA is a powerful oxidizer already at low concentrations (from 0.005% to 0.3%) (8, 9), with high efficacy against viruses, bacteria, bacterial spores, and mycobacteria, and can be used within a wide temperature range (from −40 to +20°C) (10). In comparison to HP, PAA is not inactivated by catalases (11, 12) but denatures proteins and penetrates soil loads as described for wastewater treatment procedures (9). This is particularly advantageous when decontaminating primary containment areas such as rooms for large animals.

Specific guidelines, e.g., the quantitative carrier testing (QCT) method and the European Standard 17272 for methods of airborne room disinfection by automated processes, are in place to assess the efficacy of chemical disinfectants and specify critical aspects, such as the production of the microbial inocula, the preparation of different carriers, and downstream readout procedures to calculate the log_10_ reduction of microbial pathogens or their surrogates (13–16). In fact, in most cases, commercial spore carriers (CSC) coated with 10^5^ to 10^6^ spores of Geobacillus stearothermophilus are used (3, 12, 17) and are recommended by the vendors of disinfection equipment to demonstrate the efficacy of their technology. Sophisticated methods are used in the preparation of the highly standardized CSC (18), while experimental studies are heterogeneous with respect to the volume of the inoculum, the coating procedure (drop versus smear), and the carrier material (glass or steel) of self-prepared carriers (7, 14). Therefore, we decided to adopt the QCT guideline parameters (15, 16) and used the first agreed version of the European Standard for airborne disinfection (13) to establish a robust methodology for evaluation of the efficacy of airborne disinfection with aPAA-HP under different conditions. In this study, we describe environmental factors and different carrier inoculum preparations that all influence the outcome of airborne disinfection. The widely accepted disinfection doctrine (19, 20) declares bacterial spores as the microbial organisms with the highest resistance apart from prions and coccidia. Based on this, we compared CSC with self-prepared carriers coated with Indiana vesiculovirus (VSIV), severe acute respiratory syndrome coronavirus 2 (SARS-CoV-2), murine norovirus (MNV), Mycobacterium senegalense, and spores of Geobacillus stearothermophilus and Bacillus subtilis and found significant differences in resistance between some of the tested microorganisms, especially with regard to the incubation time required for inactivation.

## RESULTS

### Feed air pressure influences particle size during aerosolization by using a Mini Dry Fog system.

We assessed the size of the aerosol droplets generated by the Mini Dry Fog device at different feed air pressures by using a laser diffraction device (*n* = 3). Thus, we observed a negative correlation resulting in mass median diameters of the generated droplets of 10.71 μm ± 0.33 μm at 180 kPa, of 7.6 μm ± 0.11 μm at 280 kPa, and of 7.46 μm ± 0.02 μm at 350 kPa and decided to run all following airborne disinfections at 350 kPa to obtain the finest and most homogenous aerosol.

### Determining the minimal effective (microbicidal) concentration of aPAA-HP.

**FIG 1:**
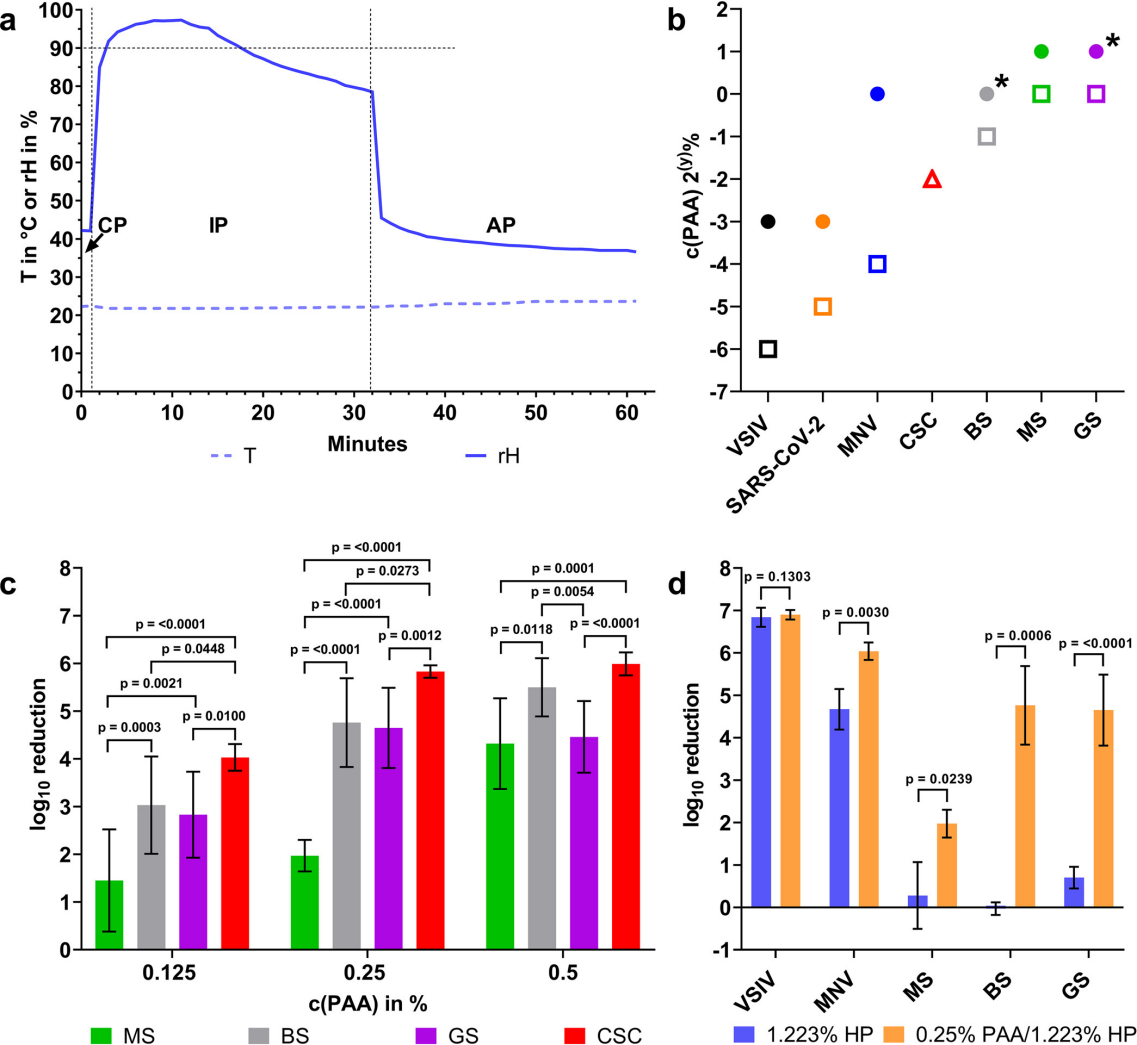
Microbiological efficacy testing of aerosolized peroxyacetic acid and hydrogen peroxide (aPAA-HP). (a) Representative graphs of relative humidity (rH) and surface temperature (T) during airborne disinfection in a biosafety cabinet. The conditioning (CP), incubation (IP), and aeration (AP) phases of the disinfection cycle can be distinguished by following the rH curve. (b) Determined minimal concentration of an aPAA-HP mixture required to achieve a ≥4-log_10_ reduction of self-prepared germ carriers of Indiana vesiculovirus (VSIV), severe acute respiratory syndrome coronavirus 2 (SARS-CoV-2), murine norovirus (MNV), *M. senegalense* (MS), and spores of B. subtilis (BS) and *G. stearothermophilus* (GS) coated as a 10-μl/cm^2^ smear (rectangles) or as a single drop (circles) and of commercial *G. stearothermophilus* spore carriers coated with a special spray method (red triangle, CSC). For simplification, only the final concentration of PAA [c(PAA), ranging from 2% to 0.0078%] in the aerosolized mixture is indicated on the *y* axis of the chart. The HP concentration in the disinfectant solution is 4.88 times higher (*n* = 7 for each tested concentration). The overall incubation time was 30 min, except for B. subtilis and *G. stearothermophilus* coated as a drop (asterisks, 60 min). (c) Bar chart with mean log_10_ reductions, pooled standard deviations as shown by error bars, and *P* values of group comparisons between MS, BS, GS, and CSC. For simplification, only the final concentration of PAA [c(PAA), ranging from 0.125 to 0.5%] in the aerosolized mixture is indicated on the *x* axis of the chart. (d) Comparison of the obtained log_10_ reductions for tested microorganisms by aerosolizing a PAA-HP mixture (final concentration, 0.25% PAA–1.223% HP) or HP alone (final concentration 1.223%). Standard deviations are shown by error bars, and *P* values between both microbicides calculated by Mann-Whitney U tests are indicated (virus, *n* = 4; bacteria, *n* = 7).

The decontamination procedure comprises three distinct phases that can be differentiated by changes of the local relative humidity (rH) level: phase 1, the conditioning phase with aerosolization of the disinfectant (until an rH of ∼90% was reached); phase 2, an incubation phase; and phase 3, an aeration and neutralization phase with room ventilation restored to remove the disinfectant completely (Fig. 1a).

Depending on the initial rH (45% to 50%), aerosolization times varied slightly (Table 1). When the target rH level was reached, the compressor was switched off for the incubation phase (IP) of 30 min. By inducing rH levels of approximately 97% at the start and no less than 80% at the end of the incubation phase, only minor condensation on the inoculated germ carrier surfaces occurred, while no condensation on other surfaces was visible. This hygroscopic effect seems to be associated with the hydrophilic properties of the proteins in the inoculum.

**TABLE 1:** Decontamination cycle parameters (means with standard deviation)

Rooms	Phase (min)^*a*^			Concn PAA-HP (%)	Disinfectant	
	CP	IP	AP		Amt (ml)	Concn (ml/m^3^)
Biosafety cabinet	0.45	30	30	Various	13 ± 1	26.8
Animal and necropsy room	20	30	30	1.2/5.8	4,242 ± 32	12.5
Laboratory	30	180	30	0.5/2.6	6,317 ± 118	9.4

a CP, conditioning phase; IP, incubation phase; AP, aeration phase.

The disinfection kinetics of the inocula prepared as a defined smear (10 μl/cm^2^) showed the highest sensitivity for the enveloped viruses VSIV and SARS-CoV-2, where marginal aerosolized peroxyacetic acid (aPAA)-hydrogen peroxide (HP) concentrations of 0.0156%/0.076% and 0.03125%/0.152%, respectively, were sufficient for >4-log_10_ reduction of infectivity. As expected, for a comparable inactivation of murine norovirus, the surrogate for nonenveloped viruses, a higher aPAA-HP concentration was required. Interestingly, CSC were only moderately resistant compared to the self-prepared germ carriers inoculated with bacterial spores and mycobacteria, which showed the highest resistance (Fig. 1b and c; see also Table S1 in the supplemental material). We selected all data points from three different aPAA-HP concentrations that did not result in a ≥4-log_10_ reduction for all three kinds of bacterial spore carriers and mycobacteria (Table S1) for statistical analysis. This revealed a highly significant difference in resistance when germ carriers with mycobacteria were compared with commercial spore carriers exposed to the same concentrations (see *P* values in Fig. 1c). Likewise, germ carriers coated with B. subtilis and *G. stearothermophilus* spores showed a variably significant higher resistance than CSC, except for one comparison of B. subtilis with CSC at 0.5% PAA-2.44% HP. When we compared self-prepared spore carriers, no significant differences in resistance were seen, except at 0.5% PAA-2.44% HP, with a significantly higher resistance of carriers coated with *G. stearothermophilus* spores (Fig. 1c). At higher concentrations, no significant differences were seen among the tested microorganisms, with sufficient or even complete inactivation of all four (Table S1).

When inocula were applied as drops of 50 μl, an unequal distribution of microorganisms occurred during air drying that was not seen in the smears. In general, for a sufficient reduction in infectivity of at least 4 log_10_ for all tested microorganisms, a significantly higher concentration of PAA-HP aerosols was necessary when the organisms were inoculated as a drop. Furthermore, no 4-log_10_ reduction was obtained during 30 min of incubation in the case of drop-inoculated bacterial spores of B. subtilis and *G. stearothermophilus.* An extension of the incubation time to 60 min was required for a sufficient reduction of B. subtilis and *G. stearothermophilus* spores (asterisks in Fig. 1b; see also Table S2).

To evaluate the main components of the disinfectant mixture, PAA and HP, concerning their individual contribution to the observed antimicrobial effect, we chose a concentration of the PAA-HP mixture that caused detectable but incomplete inactivation (0.25%/1.223%). Then, we compared the inactivation efficacy of this mixture with an aerosolized PAA-free hydrogen peroxide solution of equal strength (1.223%). For all microorganisms except VSIV, the mixture of aPAA-HP had a higher inactivation efficacy than the HP solution (Fig. 1d; see also Table S3). Especially for mycobacteria and bacterial spores, the aerosolization of HP alone at this concentration was ineffective.

### Different sensitivities of commercial and self-prepared bacterial spore carriers are due to dissimilar manufacturing techniques and carrier material properties.

**FIG 2:**
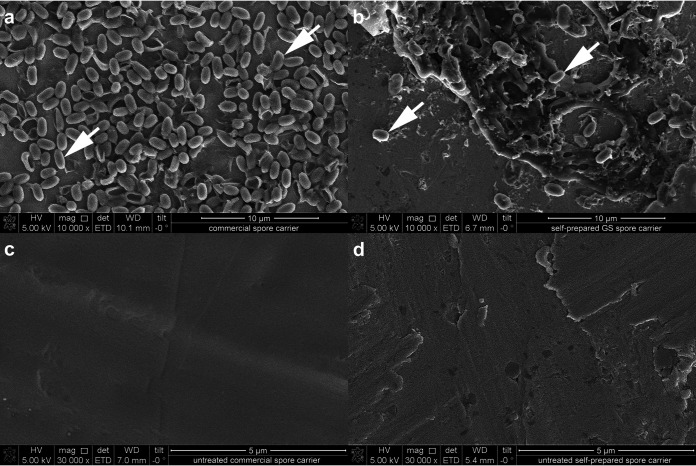
Comparison of commercial and self-prepared *G. stearothermophilus* (GS) spore carriers by scanning electron microscopy. (a) The inoculated area of a commercial spore carrier shows a uniformly distributed single layer of mostly isolated spores (arrows). (b) The inoculated area of a self-prepared spore carrier has some clearly visible scattered spores (arrows), but the majority of spores are clustered in three-dimensional aggregates. The untreated area of a commercial spore carrier shows a smooth stainless-steel surface (c), while the carrier used for the self-prepared spore carriers has multiple sharp edges and circumscribed crevices that are typical for surface quality 2B (d).

To find an explanation for the different sensitivity against aPAA-HP of the commercial and self-prepared *G. stearothermophilus* spore carriers, scanning electron microscopy was performed. As we used spores eluted from the CSC as the seed for our own spore production, we hypothesized that differences in structure or spore distribution may account for the observed differences. Commercial biological indicators inoculated by spray deposition showed a multifocal-to-confluent distribution of spores approximately in a single layer (Fig. 2a). In contrast, the self-prepared spore carriers showed a more three-dimensional distribution of multifocal islands of aggregated spores enclosed in a matrix of salt crystals and only very few scattered individual spores (Fig. 2b). Furthermore, when comparing the surfaces of the stainless-steel carriers themselves, commercial carriers were mirror finished (Fig. 2c), whereas the stainless steel of the self-prepared carriers, while still within specification (13, 16), showed a prominent relief of sharp edges and crevices (Fig. 2d). Since the scanning electron microscopy imaging of the CSC proved them to be highly artificial, we decided to exclude them from all following validation runs. Thus, for all further airborne disinfection runs, we applied the more realistic method of inoculum preparation as a smear on stainless steel carriers previously proposed by others (6).

### Successful airborne disinfection of larger rooms by aPAA-HP is mainly influenced by surface temperature differences.

Larger rooms usually have more diverse features than small biosafety cabinets, and the resulting complexity of the rooms itself can create microenvironments with locally different rH and temperature (T) values. We therefore decided to equalize such different microenvironments in larger rooms by trying to create a constant rH of 99% during the entire incubation phase of 30 min at almost all locations in our test rooms. Furthermore, we chose a concentration of 1.20% PAA and 5.86% HP for the primary containment rooms, which creates a safety margin above the minimal effective concentration for smears of all microorganisms as determined in this study (Fig. 1b).

**FIG 3:**
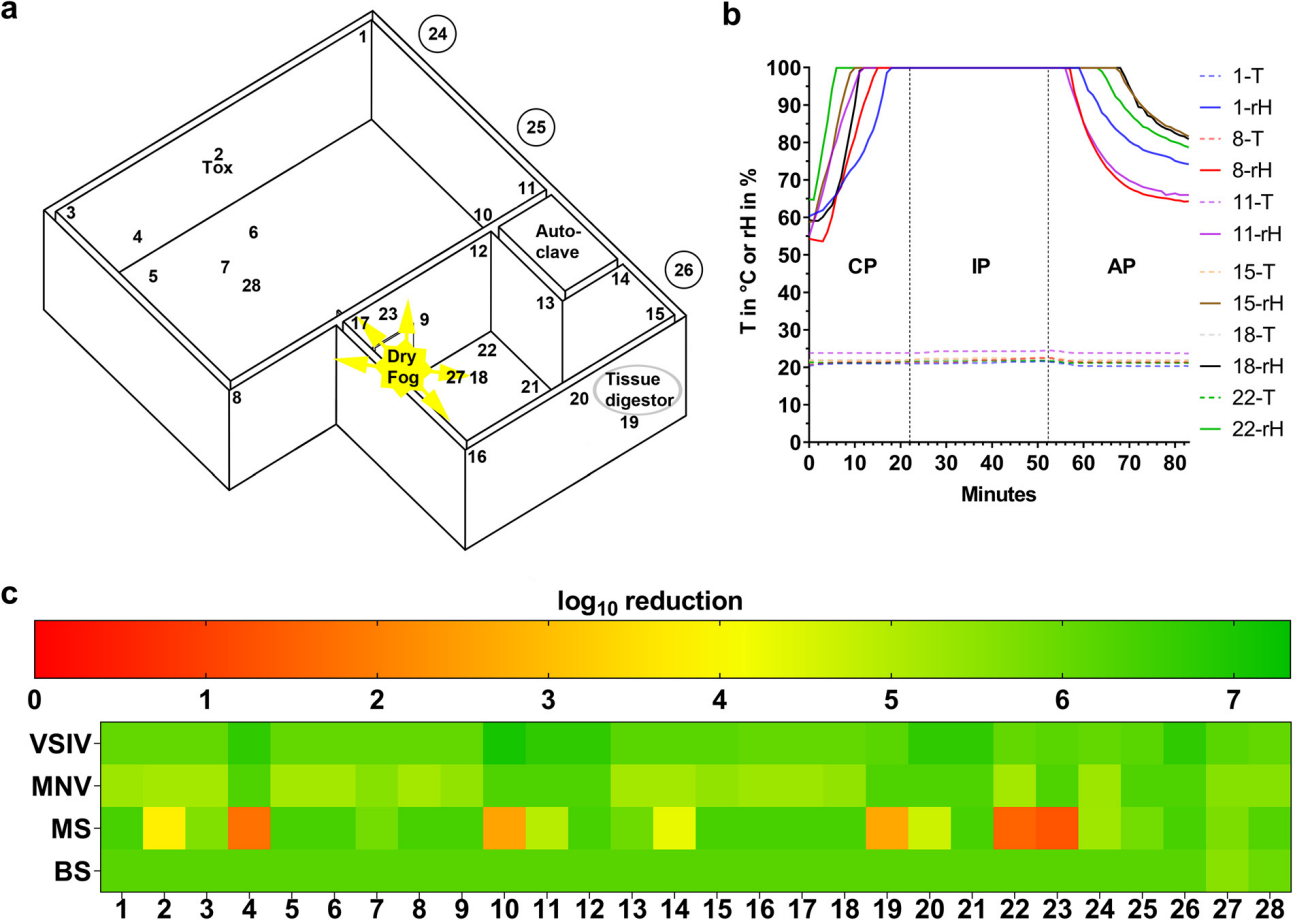
Airborne disinfection of an animal and necropsy room by aerosolizing PAA-HP. (a) Room layout with a dry fog generator placed between the two rooms with 3 nozzles spraying into the animal room (245 m^3^) and 2 nozzles into the necropsy room (95 m^3^). Numbers indicate the locations of self-prepared germ carriers in both rooms (1 to 3 and 8 to 17), on top of a ceiling lamp (4), covered by an animal husbandry device (5 and 7), in a water bowl (6), on a crane runway (18), at the bottom (19) and on a laser barrier device of a tissue carcass digestor (20), in a wastewater sewer (21), on a cutting block (22), on top of a ceiling lamp (23), 1 m deep in air exhaust ducts (24 to 26), and on the ceiling (27 and 28). Tox, toxicity control. (b) Representative rH and surface temperature graphs for six selected exposed germ carrier locations during aerosolization of 1.2%/5.86% PAA-HP. (c) Heat map of log_10_ reduction corresponding to the lower limit of the 95% confidence interval of the mean for Indiana vesiculovirus (VSIV), murine norovirus (MNV), *M. senegalense* (MS), and spores of B. subtilis (BS) coated as a 10-μl/cm^2^ smear at 28 different locations in both rooms (*n* = 3).

For an effective disinfection of a 245-m^3^ animal room and an adjacent 95-m^3^ necropsy room (Fig. 3a) that were treated simultaneously with the selected process settings (Table 1), the device has to be placed in the doorway between the two rooms with three nozzles pointing toward the animal room and two nozzles toward the necropsy room (Fig. 3a). An rH of approximately 100% was maintained during the entire incubation time of 30 min in all monitored locations (Fig. 3b). With this approach, minor condensation occurred on a few germ carriers and some horizontal surfaces of the room fittings, especially those with slightly lower temperatures.

Analysis of the germ carriers revealed complete inactivation for VSIV and MNV. Spores of B. subtilis were also sufficiently reduced at all localizations (Fig. 3c and Table S4). However, *M. senegalense* again showed the highest resistance, and its germ carriers failed to reach the required 4-log_10_ reduction at locations with elevated surface temperatures, e.g., on illuminated ceiling lamps at locations 4 and 23 (in total, there were 13 fluorescent tubes in both rooms) and on the wall and floor next to an autoclave or a tissue digestor at location 10 or 19, respectively (Fig. 3a and c and Table S4). The data loggers, necropsy equipment, room surfaces, and electronic components remained unaffected during all testing and validation runs.

### Reduced PAA concentration with extended incubation time results in efficient disinfection of a fully equipped laboratory.

**FIG 4:**
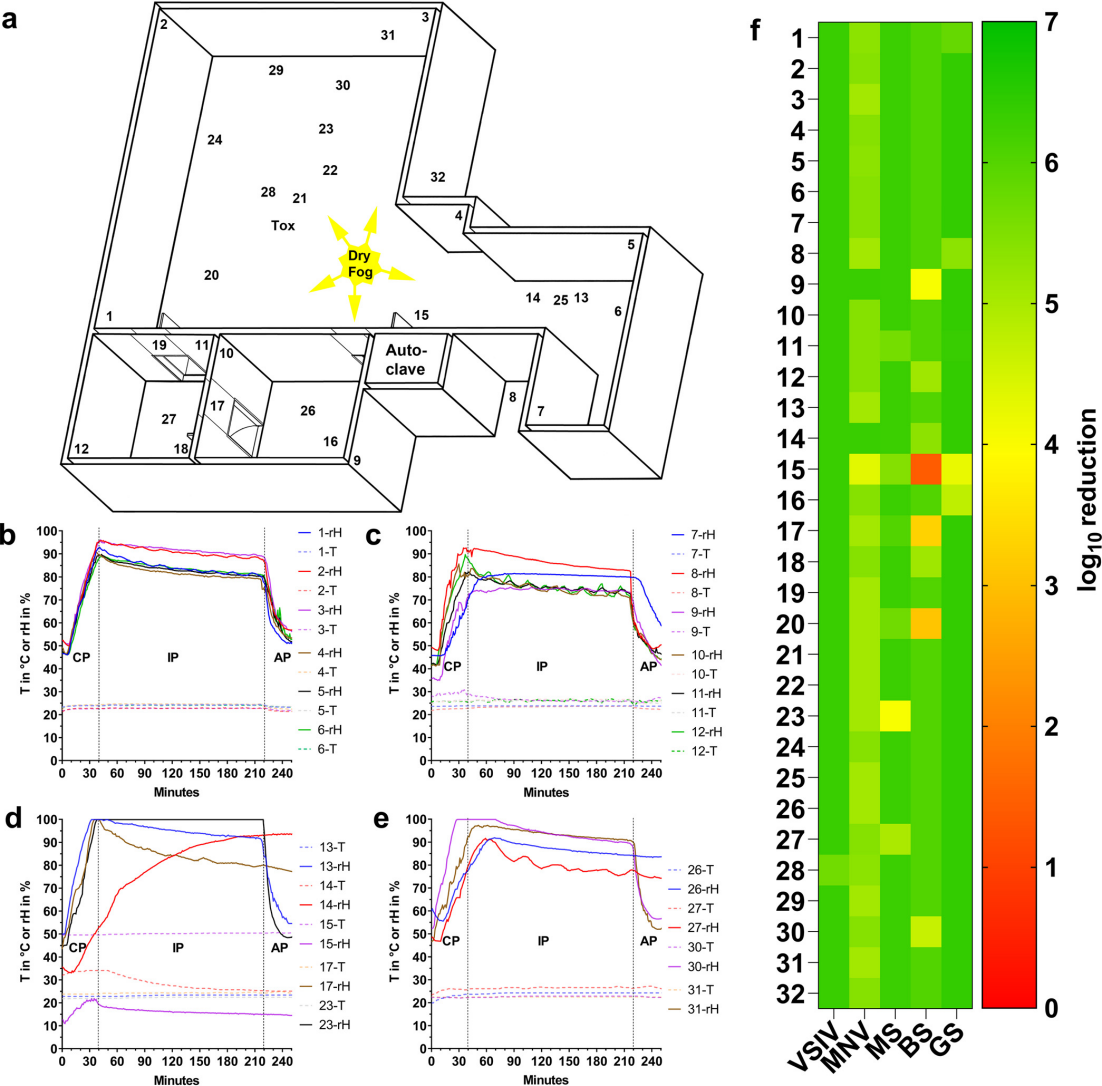
Airborne disinfection of a complex laboratory by aerosolizing PAA-HP. (a) Room layout of the laboratory with two side rooms and a material lock. The dry fog device was placed in the center of the laboratory, with 5 nozzles spraying to the far corners of the room. Numbers indicate the positions of germ carriers in the main laboratory (1 to 6), in a material airlock (7), in a technical space above the material airlock (8), in side rooms (9 to 12), inside a live-cell imaging microscope (13), on top of a ceiling lamp (14), at the edge of an autoclave (in stand-by mode) (15), under an ultracentrifuge (16), on top of a liquid-nitrogen tank (17), behind a door (18), 1 m deep in an air exhaust duct (19, 26, and 28), on top of a ceiling lamp (20), in a storage cabinet (21 and 30), under a centrifuge (22), within a centrifuge rotor (23), inside a personal computer with the fan running (24), on the ceiling (25 and 27), in the technical space of a refrigerator (29), behind the wall covering of the laboratory sink (30), behind a −20°C freezer (31), and 1 m deep in another air exhaust duct (32). (b to e) Representative rH and surface temperature graphs for selected exposed germ carrier locations during aerosolization of 0.5%/2.44% PAA-HP in the main laboratory. (f) Heat map of log_10_ reduction corresponding to the lower limit of the 95% confidence interval of the mean for Indiana vesiculovirus (VSIV), murine norovirus (MNV), *M. senegalense* (MS), and spores of B. subtilis (BS) and *G. stearothermophilus* (GS) coated as a smear at 32 different locations (*n* = 3).

No damage was observed during testing and validation of the above-described airborne disinfection protocol, except for minor corrosion on brass coatings. In line with the observation that an extended incubation time resulted in more sufficient inactivation (Fig. 1b), we observed that an extension of the incubation time from 30 to 180 min can compensate for a reduction of the aPAA-HP concentration (from 1.20%/5.86% to 0.5%/2.44%) and a lower rH of 90% induced by the cycle parameters described in Table 1 within a fully equipped laboratory of 675 m^3^ (Fig. 4a and Table 1). The prolonged disinfection cycle with lower rH levels than in previous experiments (Fig. 4b to e) resulted in even better inactivation of the tested germ carriers (Fig. 4f; see also Table S5). An elevated surface temperature of 50°C at an autoclave door (location 15, Fig. 4d) prevented rehydration of the inoculated germ carriers placed there. We assume that the measured inactivation of the test microorganisms at this position resulted mainly from the elevated temperature, which was considered high enough to inactivate all tested organisms except spores of B. subtilis. A similar effect was seen at the beginning of the process for ceiling lamps (locations 14 and 20; in total, there were 29 fluorescent tubes in the three rooms) that were then switched off in the middle of the conditioning phase (CP), resulting in a decline in temperature (middle of CP [20 min], 33.7°C, to end of IP [220 min], 25.3°C) and a remarkable rise in rH (from 38.2% to 93.0%) during the incubation phase (location 14 in Fig. 4a, red line in Fig. 4d). This resulted in a satisfactory reduction of all tested microorganisms at this location (>4-log_10_ reduction) (Fig. 4f; Table S5). That very low temperatures can also have a negative influence was evident on the outer cover of a liquid-nitrogen tank (location 17), where sufficient reduction of the B. subtilis spores was narrowly missed. In general, the extended decontamination cycle with 0.5% PAA and 2.44% HP was more effective in terms of the inactivation of mycobacteria than the shorter runs, supporting our hypothesis of the importance of sufficient rehydration and penetration of the inoculum. All data loggers, personal computers, freezers, centrifuges, and the live-cell imaging microscope in the laboratory were unaffected by a PAA-HP dry fogging.

## DISCUSSION

Airborne disinfection with low concentrations of a mixture of PAA-HP as ultrafine aerosols resulted in a highly effective and rapid decontamination of high-containment areas. While HP- and formaldehyde-based procedures are time consuming (between 12 and 48 h), our method achieved at least a ≥4-log_10_ reduction of infectivity in 1 to 4 h. We showed in aPAA-HP sensitivity experiments that achieving a ≥4-log_10_ reduction depends on the concentration of the disinfectant mixture, the relative humidity, and the incubation time. On the other hand, the incubation or contact time of the aerosols with vertical surfaces is mainly driven by particle size. In general, smaller droplets stay airborne longer, e.g., 5-μm particles are airborne for ∼67 min, 10-μm particles for ∼17 min, and 20-μm particles for ∼4 min (21, 22). Therefore, we selected a feed air pressure of at least 350 kPa to obtain aerosols smaller than 7.5 μm in diameter.

In line with published data for sensitivity testing of microorganisms against biocides (19), viruses were highly sensitive against PAA-HP aerosols, while mycobacteria and spore-forming bacteria were least sensitive. We show that the enveloped SARS-CoV-2 was readily inactivated even by an extremely low concentration of aPAA-HP, proving the applicability of airborne disinfection with aPAA-HP also for the ongoing pandemic situation.

Furthermore, we observed a significantly higher resistance of self-prepared mycobacteria and spore carriers compared to that of CSC. This is most likely caused by the surface finish of stainless-steel germ carriers and the coating method. These commercial spore carriers are widely used in health care and pharmaceutical industries to monitor the performance of gaseous or liquid disinfection technologies (6, 12, 23). Disinfection procedures are usually considered successful when a 6-log_10_ reduction of infectivity in such a spore monolayer created by spray deposition (18) is achieved. However, our results indicate that this is misleading. The measured reduction of spores prepared in accordance with quantitative carrier testing requirements (multilayered and covered by a matrix) (13, 14) was much lower. We carefully reviewed the existing literature and found few reports describing that commercial spore carriers were easier to inactivate than self-prepared carriers. Krishnan and colleagues reported that after 30 min of PAA dry fogging, commercial *G. stearothermophilus* spore carriers were inactivated, while self-prepared virus and Bacillus atrophaeus spore carriers required 1 h or even overnight exposure for complete inactivation (7). Similar results were seen after fumigation with 5% HP, when commercial B. atrophaeus spore carriers were inactivated, while all carriers coated with Mycobacterium tuberculosis still showed growth (23). Even Gram-positive Staphylococcus aureus bacteria were more resistant against vaporized HP than commercial *G. stearothermophilus* spore carriers (12). All these results were generated with different decontamination technologies but are in alignment with our results. Therefore, we recommend avoiding the use of commercial spore carriers for the validation of disinfection in high-containment environments where microorganisms other than viruses are handled. Instead, quantitative carrier testing with different classes of surrogate microorganisms representative of the pathogens that are in fact present in the facility should be performed.

Another important aspect is the preparation of the biological indicator itself. Disinfectant testing lacks harmonized and globally accepted quantitative carrier testing requirements. Accordingly, the existing literature (7, 14) varies enormously with respect to the volume of inoculum (e.g., 10 μl versus 50 μl), the coating method (drop versus smear), and the carrier material (glass versus steel). To highlight only one of these aspects, we focused on the way how the inoculum is deposited on the carrier surface. We decided to distribute our microorganisms more homogenously as a smear, because this mirrors a contaminated surface after a wet cleaning step, which should always be performed prior to airborne disinfection. One constraint in our own inoculum preparation was the addition of polysorbate 80 to the mycobacterial suspensions. However, this was the only feasible way to create a homogenized smear by breaking the surface tension and avoiding reaggregation of mycobacteria with consecutive clump formation. We want to point out that the addition of polysorbate 80 might have skewed the log_10_ reduction but without an obvious increase in susceptibility to PAA and HP as indicated earlier. In our view, airborne disinfection in general can supplement, but never replace, thorough mechanical cleaning of contaminated surfaces.

The electron microscopy images of stainless-steel carriers inoculated with a single drop reflected the most likely scenario of a contaminated surface before wet cleaning and emphasized the three-dimensional aspect of spore aggregation. Thus, under our conditions, we required much higher concentrations of the PAA-HP mixture to achieve the targeted ≥4-log_10_ reduction when viruses and mycobacteria were used as drops on self-prepared biological indicators and even failed to meet that threshold in the case of bacterial spores. We hypothesize that the “coffee ring effect” (24) has a major impact on enhancing the resistance of any biological indicator. When the fluid evaporates during drying of the drop, suspended particles (such as microorganisms) are deposited along the perimeter of the drop (25) and form multilayered aggregates. Therefore, inoculating carrier surfaces with clearly visible drops is out of the scope for airborne disinfection methods as had been pointed out in a German guideline by the Robert Koch Institut as early as 1994 (25). It is obvious that all these variables of biological indicator characteristics influence the outcome of any disinfection efficiency testing. Therefore, the recently published European standard for airborne room disinfection by automated processes provides harmonized protocols and is thus essential for the generation of comparable data sets (13).

To increase the portability of the dry fogging method, we decided to use a mobile compressor instead of a permanently installed compressed air supply line. At the same time, using a compressor in the treated room itself avoids increasing the air pressure in the room, as no additional air is introduced from outside. This prevents damage to the infrastructure or interference with negative-pressure environments. Besides the described influence of the carrier preparation, we further showed that the building environment is of equal importance for a successful disinfection and must be assessed carefully. Especially for complex room layouts with heat sources (e.g., autoclaves in adjacent rooms, activated ceiling lamps, and incubators), room surface temperatures are highly variable and can range from 20°C to 50°C. These temperature gradients create local microenvironments which inhibit the uniform distribution of the aerosolized disinfectant throughout the treated space, as indicated by a measurably lower relative humidity, and lead to a reduced interaction of the disinfectant with affected surfaces. Since minor condensation and rehydration of inoculated surfaces by the aPAA-HP solution is a major factor for successful disinfection, this physical effect results in insufficient microbial killing at these locations. Even smaller temperature differences are of importance, as we noted a relevant difference in inactivation efficacy for temperature gradients of around 10°C (see Tables S4 and S5 in the supplemental material). This observation might be due to thermal boundary layers and/or convective airflow within enclosed rooms (26). Thus, electrical equipment, including light fixtures, should be switched off during the disinfection procedure if possible. However, if complex electronic devices such as personal computers must be decontaminated, active interior circulation of the aerosolized disinfectant by the cooling fan of the unit is most effective for parts without large temperature gradients.

The importance of sufficient rehydration for a certain time was shown for *M. senegalense*, which was the most resistant microbe in the short process at high relative humidity (Fig. 1b and 3c), in contrast to the generally accepted sensitivity hierarchy (or “tenacity pyramid”), where some authors see mycobacteria as more sensitive to biocides than small nonenveloped viruses or bacterial spores (19). The most likely reason for this enhanced resistance is a delayed and insufficient rehydration (23) of the mycobacterial aggregates under these conditions. The lipid-rich waxy cell wall facilitates the tight attachment to surfaces and causes resistance against disinfectants (27). When the exposure time in the laboratory was extended to 180 min, all mycobacteria on germ carriers were successfully inactivated, and the spores of B. subtilis were the most resistant, as initially expected. This indicates a major influence of the incubation time and suggests that a further reduction of the aPAA-HP concentration is possible without compromising inactivation efficacy, particularly for viruses. For this reason and because of its portability and the ease of monitoring, its most important parameter (relative humidity), this airborne disinfection method is not only suitable for use in laboratory and animal spaces but can also be easily adapted for use in other premises and even public transportation.

## MATERIAL AND METHODS

### Dry fogging and measuring equipment.

Two portable dry fogging systems, Mini Dry Fog and Dry Fog 2-S (Mar Cor, Heerlen, The Netherlands), were used to generate ultrafine disinfectant aerosols. Working solutions of disinfectant were obtained by dilution of a stock mixture that contained 4.5% PAA and 22% HP by volume in deionized water. A mobile air compressor (model C330/03; Gentilin, Trissino, Italy) was used to supply compressed air. The compressor was placed within the treated space and was remotely operated by radio-controlled sockets (model ZAP 5LX; Etekcity, Shenzen, China).

Portable measuring devices with data loggers (model 635-2; Testo, Lenzkirch, Germany) as well as a permanently installed rH and T measurement system (model Saveris H2 D; Testo) were used for wireless monitoring during decontamination. The rH probes were calibrated in-house by two-point calibration. A variation of ±5% of rH over the measuring range of each device was considered acceptable (28). The measured values were stored and analyzed after each trial by using the vendor software. To measure surface temperatures, a thermal imaging camera (model 882; Testo, Lenzkirch, Germany) was used.

### Aerosol particle size measurement.

Aerosolized deionized water was used for particle-size measurements instead of a mixture of PAA-HP to avoid damage to the laser diffraction device (MasterSizer MS20; Malvern Instruments, Worcestershire, UK). The mass mean diameter in microns was determined by using a conventional Fourier lens with a focal length of 100 mm. Feed air pressures of 180, 280, and 350 kPa (*n* = 3) in combination with the Mini Dry Fog system were used to assess the influence of this parameter on the droplet sizes.

### Decontamination setup.

All experiments were conducted within the containment facilities of the Friedrich-Loeffler-Institut (FLI), Insel Riems, Germany. Reproducible airborne disinfection protocols were established in a biosafety cabinet, an animal room, a necropsy room, and a laboratory.

**(i) Decontamination within a biosafety cabinet (0.5-m^3^ total volume).** The Mini Dry Fog system was used for the inactivation experiments within a class 2 biosafety cabinet (0.5-m^3^ interior volume). To determine the minimal effective concentration resulting in a ≥4-log_10_ reduction of infectivity of the tested microorganisms (defined as the minimal effective concentration), the PAA-HP mixture containing 4.5% PAA and 22% HP was used at concentrations ranging from 0.0156%/0.076% to 2.00%/9.76%, with ≥7 germ carriers for each tested concentration, as summarized in Tables S1 and S2 in the supplemental material. Germ carriers were inoculated with VSIV and MNV in cell culture supernatant, *M. senegalense* in phosphate-buffered saline (PBS) with 1% polysorbate 80, and spores of B. subtilis and *G. stearothermophilus* in PBS either as a defined smear (50 μl overall, 10 μl/cm^2^) or as a single drop of 50 μl. The infectious particle number per 50 μl was as follows: 2.81 × 10^8^ (VSIV), 3.37 × 10^5^ (SARS-CoV-2), 2.81 × 10^7^ (MNV), 1.58 × 10^7^ (*M. senegalense*), 6.60 × 10^6^ (B. subtilis), and 9.15 × 10^5^ (*G. stearothermophilus*). For comparing the microbiocidal activity of the main components, a PAA-HP mixture of 0.25%/1.223% and an aqueous solution of only HP at a concentration of 1.223% (prepared from a 30% stock solution) was aerosolized (7 germ carriers for bacteria, 4 germ carriers for viruses) (see Table S3). To avoid visible condensation on surfaces, rH levels of approximately 97% at the start and no less than 80% at the end of the incubation phase were set for the exposure of bioindicators to PAA-HP aerosols.

**(ii) Decontamination of animal and necropsy rooms.** All room surfaces are coated with epoxy resin, and the rooms are fitted with animal husbandry and necropsy equipment made of stainless steel. The animal room (66-m^2^ floor area, 245-m^3^ total volume) and the adjacent necropsy room (26 m^2^, 95 m^3^) that also contained an overhead crane and a tissue digestor were decontaminated together. Both rooms are equipped with five air inlets and three air exhaust ducts.

Twenty-eight bioindicators coated with VSIV, MNV, *M. senegalense* mycobacteria, and spores of B. subtilis as a defined smear (10 μl/cm^2^) and six mobile data loggers were placed at predefined positions (Fig. 3a and b). A Dry Fog 2-S system with 5 horizontal nozzles was placed in the doorway between the rooms (Fig. 3a) and filled with a mixture of disinfectant with final concentrations of 1.20% PAA and 5.86% HP. The decontamination cycle was executed as described in Table 1.

**(iii) Decontamination of a laboratory.** The laboratory suite comprised a main room, two side rooms, and a material airlock with a total floor surface and room volume of 186 m^2^ and 675 m^3^, respectively, equipped with 10 air inlets and seven air exhaust ducts. Besides standard laboratory equipment (e.g., microscopes and biosafety cabinets), a live-cell imaging microscope and a fluorescence-activated cell sorter remained in the room during the disinfection process. A Dry Fog 2-S unit with 5 horizontal nozzles was placed in the center of the main room (Fig. 4a) and filled with a mixture of disinfectant with final concentrations of 0.5% PAA and 2.44% HP. Bioindicators coated with VSIV, MNV, *M. senegalense*, and spores of B. subtilis and *G. stearothermophilus* as a defined smear (10 μl/cm^2^) at 32 locations as well as 21 data loggers were placed at predefined positions (Fig. 4a to e), and a standard decontamination cycle was run (Table 1).

### Germ carrier preparation and analysis.

**(i) Testing and readout of commercial spore carriers.** Apex Ribbons (Mesa Labs, Chassieu, France) coated with 2.1 × 10^6^ *G. stearothermophilus* spores (ATCC 12980) with an indicated thermal resistance (*D* value) of 1.7 min in 2 mg/liter gaseous hydrogen peroxide were removed from their Tyvek pouches immediately prior to PAA fogging in the initial PAA sensitivity tests. After each cycle, the spore-coated end of a bioindicator (approximately 1 cm long) was cut off with sterile scissors and directly placed into a tube containing 1 ml Trypticase soy broth (TSB; Carl Roth, Karlsruhe, Germany). To lift the spores from the ribbons, the ribbons were scrubbed with a pipette tip, and the tube was vortexed twice for 30 s.

An untreated ribbon was used as positive control. The spores were titrated in 96-well U-bottom plates and incubated at 60°C for 7 days, and the most probable number (MPN) method (29) was used to quantitate the success of decontamination. A positive control remained untreated in the laboratory during each decontamination cycle.

**(ii) Self-prepared germ carriers.** Stainless-steel carriers (GK Formblech, Berlin, Germany) were procured with an EN 10088-2 quality 2B surface treatment on both sides. They were rectangular, measuring 16 by 60 mm with a thickness of between 1.2 and 1.5 mm depending on the lot. Carriers were immersed in 5% Decon 90 solution (Decon Laboratories, Hove, UK) for 60 min, in 70% ethanol for 15 min, rinsed in demineralized water, and air dried in a biosafety cabinet (BSC). All carriers were sterilized with dry heat at 180°C for at least 2 h. All carriers were reused multiple times until pitting of the stainless-steel surfaces became evident.

**(iii) Germ carrier coating and recovery of microorganisms.** Stainless steel carriers were coated with 50 μl of each microorganism from thawed stock solutions, as described elsewhere (6, 25), either spread out with a horizontally held pipette tip (10 μl per cm^2^) or as one drop of 50 μl. With spore and virus suspensions, a homogeneous coating was obtained, but hydrophobic mycobacteria formed visible clots. To establish a homogenous distribution of mycobacteria, three washes with PBS followed by resuspension in PBS with 1% polysorbate 80 and pipetting up and down (10 times) through a 0.4-mm (27 gauge) cannula were performed. The coated germ carriers were air dried in a BSC.

After PAA fogging, germ carriers were collected and washed with 1 ml of bacterial growth medium for spores and mycobacteria or with cell culture medium for viruses. The carriers were placed upright in 12-well plates and washed 10 times by manually up-and-down pipetting of 750 μl of the liquid for viruses and 1,000 μl for spores and mycobacteria. Carriers with spores and mycobacteria were also scrubbed with a horizontally held pipette tip.

**(iv) Controls.** Drying controls were used in each trial and were stored in closed boxes immediately after air drying.

Stainless steel carriers coated with cell culture supernatant or bacterial growth medium served as toxicity controls. The use of disinfectant neutralizer was avoided, since all stocks had titers high enough to allow the verification of a reduction of infectivity of >4 log_10_, which was defined as successful decontamination (14).

**(v) Cell lines and virus propagation.** The cells were routinely propagated in the presence of 10% fetal bovine serum (FBS). An overview of the amplification conditions of the viruses is given in Table 2. Virus stocks were prepared by inoculating a >90% confluent layer of cells followed by incubation at 37°C and 5% CO_2_ until a prominent cytopathic effect developed. Virus was harvested by freezing and thawing of cells to release viral particles. After thawing, the supernatant was centrifuged at 2,000 × *g* for 10 min and stored in aliquots at −80°C until use.

**TABLE 2:** Viruses, cells, incubation times, and titers used for inactivation experiments

Virus	Cell line	Culture medium^*a*^	Incubation time (h)	Stock titer (TCID_50_/ml)
MNV	RAW 264.7 (CCLV-RIE 0996)^*b*^	MEM plus 2% FBS	18–24	5.62 × 10^8^
SARS-CoV-2	Vero E6 (CCLV-RIE 0929)	MEM (Hanks’ salts) plus MEM (Earle’s salts) plus nonessential amino acids plus 10% FBS	48–52	6.74 × 10^6^
VSIV	LFBK-αVβ6 (CCLV-RIE 1419)	DMEM plus 2% FBS	12	5.62 × 10^9^

a MEM, minimal essential medium; DMEM, Dulbecco modified Eagle medium.

b CCLV-RIE, Collection of Cell Lines in Veterinary Medicine, FLI Riems, Greifswald, Germany.

**(vi) Virus infectivity assay.** To estimate viral titers, endpoint titration was performed in 96-well plates with an evaluation of the cytopathic effect after 5 to 6 days. Fifty percent tissue culture infectious doses (TCID_50_) per milliliter were calculated by the Spearman-Kärber method (30). The titers of the virus stocks are shown in Table 2. The limit of detection of infectivity was 1.8 log_10_ TCID_50_/ml. In some cases, PAA condensation on the carrier surface caused nonspecific cytotoxicity and raised the limit of detection to 2.8 log_10_ TCID_50_.

**(vii) *G. stearothermophilus*.** From the commercially acquired Apex Ribbons spore carriers, *G. stearothermophilus* was isolated and grown on brain heart infusion agar (Carl Roth) in 250-ml bottles at 60°C. After 96 h, the predominantly sporulated microorganisms were washed off, and the colony material was resuspended and washed three times in phosphate-buffered saline (PBS) after centrifugation at 3,200 × *g* for 10 min before aliquoting and storage at −20°C. The sporulation rate was determined thermally. For this purpose, an aliquot was heated for 10 min at 100°C (31), which inactivated vegetative forms, so that in the subsequent determination of growth by using the most probable number (MPN) method via plate counting or dilution method, only the remaining germinating spores were detected with a stock count of 1.83 (± 0.006) × 10^7^ MPN/g.

**(viii) B. subtilis.** B. subtilis spores (an environmental isolate kindly provided by the State Office for Agriculture, Food Safety and Fishery of Mecklenburg-Western Pomerania, Rostock, Germany) were chosen as a representative surrogate for Bacillus anthracis (32, 33). Spores were harvested similarly to the spores of *G. stearothermophilus*. Only the temperatures for cultivation (37°C instead of 60°C) and for determination of the number of spores (80°C instead of 100°C) differed. Furthermore, only the plate counting method was used for determination of CFU per gram. The stock count was 1.32 (± 0.01) × 10^8^ CFU/g.

**(ix) *M. senegalense*.** *M. senegalense* (DSM 110653, kindly provided by the Institute of Bacteriology and Mycology, Faculty of Veterinary Medicine and Institute for Medical Microbiology and Epidemiology of Infectious Diseases, University Hospital of Leipzig, Leipzig University, Leipzig, Germany) is a nontuberculous member of the Mycobacterium fortuitum complex with high veterinary but uncertain human-pathogenic relevance (34–36). Members of the M. fortuitum complex have been used before for disinfectant testing (37) and for room fumigation procedures as surrogates for M. tuberculosis (38). *M. senegalense* was cultured on Trypticase soy agar (Carl Roth), rinsed off and washed in PBS, and frozen in 10% glycerol-PBS solution in 1-ml aliquots. The concentration of mycobacteria was 3.15 (± 0.375) × 10^8^ CFU/g.

**(x) Decontamination efficacy and statistical analysis.** The efficacy of aPAA-HP dry fog decontamination was assessed by calculating the log_10_ reduction of infectivity by subtracting the logarithmic values of TCID_50_ per milliliter, CFU per milliliter, or MPN per milliliter of decontaminated germ carriers from the untreated positive controls. For experiments in the BSC, the data for each germ carrier location are expressed as a mean (*n* = 7) with pooled standard deviation (pSD) of both means using the following formula (39):

$$s=\sqrt{\frac{\left(n_{1}\;-\;1\right)s_{1}^{2}\;+\;\left(n_{2}\;-\;1\right)s_{2}^{2}}{n_{1}\;+\;n_{2}\;-\;1}}.$$

A two-way analysis of variance (ANOVA) with Tukey’s *post hoc* test was performed for mean log_10_ reductions on commercial spore carriers and selected self-prepared germ carriers prepared as smears during testing of the minimal effective (microbicidal) concentration of aPAA-HP. For pairwise comparisons of the inactivation efficacy of a PAA-HP concentration of 0.25%/1.223% against an HP solution (1.223%), the nonparametric Mann-Whitney U test was applied. A *P* value of ≤0.05 was considered significant.

For room decontamination experiments, where one bioindicator served as a positive control and one bioindicator of each surrogate was tested at each location, the variance was zero. Therefore, the logarithmic values of the positive control and decontaminated carrier were subtracted, and the mean with standard deviation from three replicated experiments was calculated. Statistical analyses, figures for rH and T, and heat maps were created with Prism version 7.0.4 (GraphPad Software, San Diego, CA, USA).

**(xi) Scanning electron microscopy.** Investigations of the carrier surfaces and the morphology of the coated microorganisms were conducted by high-resolution scanning electron microscopy (SEM) using a Quanta three-dimensional (3D) field emission gun (FEI Co., Hillsboro, OR, USA). The samples were placed on SEM specimen plates using carbon tape and coated with a thin layer of platinum by magnetron sputtering (HVD, Dresden, Germany) to achieve a conducting surface. The SEM images were generated in high-vacuum mode using an acceleration voltage of 5 keV, a working distance of 6 to 10 mm and electron beam currents of 12 pA determined with an Everhart Thornley detector.

## Supplementary Material

Supplemental file 1
