# Supplementary material for: Airborne Disinfection by Dry Fogging Efficiently Inactivates Severe Acute Respiratory Syndrome Coronavirus 2 (SARS-CoV-2), Mycobacteria, and Bacterial Spores and Shows Limitations of Commercial Spore Carriers
Source: Appl Environ Microbiol. 2021 Jan 15;87(3):e02019-20. doi: 10.1128/AEM.02019-20 (PMC7848922; doi:10.1128/AEM.02019-20)
Supplement: Supplemental file 1 [file AEM.02019-20-s0001.pdf]

Applied and Environmental Microbiology – Supplemental Material

“Airborne disinfection by dry fogging efficiently inactivates SARS-CoV-2, mycobacteria and bacterial spores and shows the limits of commercial spore carriers for process control”

Table S1. Log_{10} reduction of infectivity (means with pooled standard deviation) of germ carriers coated with microorganisms as 10 $\mu l/cm^2$ after aerosolization of variable PAA concentrations and 30 min incubation time.

c(PAA) in %	VSIV	SARS-CoV-2	MNV	MS	BS	GS	CSC
**0**	1.71 ± 0.34	nd	nd	nd	nd	nd	nd
**0.0078**	3.76 ± 0.47	nd	nd	nd	nd	nd	nd
**0.0156**	6.34 ± 0.64	3.04 ± 0.44	nd	nd	nd	nd	nd
**0.0313**	7.19 ± 0.26[a]	4.60 ± 0.37	3.91 ± 0.28	nd	nd	nd	nd
**0.0625**	6.61 ± 0.08[a]	4.68 ± 0.18[a]	6.09 ± 0.11[a]	nd	nd	nd	2.01 ± 0.48
**0.1250**	nd	nd	nd	1.45 ± 1.07	3.03 ± 1.02	2.83 ± 0.90	4.03 ± 0.28
**0.2500**	nd	nd	nd	1.97 ± 0.33	4.76 ± 0.93	4.65 ± 0.84	5.83 ± 0.13[a]
**0.5000**	nd	nd	nd	4.32 ± 0.95	5.50 ± 0.61	4.46 ± 0.75	5.99 ± 0.24[a]
**1.0000**	nd	nd	nd	5.98 ± 0.30[a]	5.46 ± 0.47	5.31 ± 0.60	5.94 ± 0.16[a]

c(PAA), concentration of peroxyacetic acid; VSIV, Indiana vesiculovirus; SARS-CoV-2, severe acute respiratory syndrome coronavirus 2; MNV, murine norovirus; MS, *M. senegalense*; BS, *B. subtilis* spores; GS, *G. stearothermophilus* spores; CSC, commercial spore carriers coated with GS spores; [a], complete inactivation; nd, not done.

Table S2. Log_{10} reduction of infectivity (means with pooled standard deviation) of germ carriers coated with microorganisms as a drop after aerosolization of variable PAA concentrations and 30 or 60 min incubation time.

c(PAA) in %	VSIV	SARS-CoV-2	MNV	MS	BS	GS	CSC
**0.0625**	3.81 ± 0.42	nd	nd	nd	nd	nd	2.01 ± 0.48
**0.1250**	5.67 ± 0.27	5.05 ± 0.16[a]	nd	nd	nd	nd	4.03 ± 0.28
**0.2500**	7.04 ± 0.28[a]	5.00 ± 0.23[a]	2.62 ± 0.20	2.62 ± 0.54	3.81 ± 0.77	2.53 ± 0.53	5.83 ± 0.13[a]
**0.5000**	7.05 ± 0.25[a]	nd	4.05 ± 0.30	4.21 ± 0.35	3.52 ± 0.42	3.37 ± 0.36	5.99 ± 0.24[a]
**1.0000**	7.42 ± 0.17[a]	nd	4.57 ± 0.50	4.10 ± 0.63	4.21 ± 0.63	4.42 ± 0.82	5.94 ± 0.16[a]
**2.0000**	nd	nd	nd	4.93 ± 0.49	2.73 ± 0.17	3.28 ± 0.35	nd
**1.0000**[b]	nd	nd	nd	nd	5.97 ± 0.64	3.23 ± 0.32	nd
**2.0000**[b]	nd	nd	nd	nd	nd	5.72 ± 0.24	nd

c(PAA), concentration peroxyacetic acid; VSIV, Indiana vesiculovirus; SARS-CoV-2, severe acute respiratory syndrome coronavirus 2; MNV, murine norovirus; MS, *M. senegalense*; BS, *B. subtilis* spores; GS, *G. stearothermophilus* spores; CSC, commercial spore carriers coated with GS spores; [a], complete inactivation; [b], 60 min incubation time; nd, not done.

Table S3. Log_{10} reduction of infectivity (means with pooled standard deviation) of germ carriers coated with microorganisms as a 10 µl/cm^2 smear after aerosolization of 1.223% HP or 0.25% PAA and 1.223% HP.

	1.223% HP	0.25% PAA/1.223% HP
**VSIV**	6.84 ± 0.22[a]	6.90 ± 0.11[a]
**MNV**	4.67 ± 0.48	6.04 ± 0.20[a]
**MS**	0.28 ± 0.79	1.97 ± 0.33
**BS**	-0.03 ± 0.15	4.76 ± 0.93
**GS**	0.70 ± 0.25	4.65 ± 0.84

VSIV, Indiana vesiculovirus; MNV, murine norovirus; MS, *M. senegalense*; BS, *B. subtilis* spores; GS, *G. stearothermophilus* spores; [a], complete inactivation.

Table S4. Log_{10} reduction of infectivity as mean and standard deviation (SD) of germ carriers with surface temperature (mean and SD) at distinct locations in an animal and necropsy room after aerosolization of 1.20%/5.86% PAA/HP.

GC	VSIV	MNV	MS	BS	T in °C
**1**	6.33 ± 0.29[a]	5.39 ± 0.10[a]	6.49 ± 0.09[a]	6.27 ± 0.09[a]	21.27 ± 0.12
**2**	6.33 ± 0.29[a]	5.72 ± 0.54[a]	5.15 ± 1.41	6.27 ± 0.09[a]	21.27 ± 0.23
**3**	6.33 ± 0.29[a]	5.72 ± 0.54[a]	6.16 ± 0.54	6.27 ± 0.09[a]	21.50 ± 0.36
**4**	7.00 ± 0.29[a]	6.39 ± 0.10[a]	2.26 ± 0.50	6.27 ± 0.09[a]	33.13 ± 0.31
**5**	6.33 ± 0.29[a]	5.72 ± 0.54[a]	6.49 ± 0.09[a]	6.27 ± 0.09[a]	23.47 ± 0.31
**6**	6.33 ± 0.29[a]	5.72 ± 0.54[a]	6.49 ± 0.09[a]	6.27 ± 0.09[a]	20.97 ± 0.21
**7**	6.33 ± 0.29[a]	6.05 ± 0.48[a]	6.23 ± 0.41	6.27 ± 0.09[a]	21.30 ± 0.26
**8**	6.33 ± 0.29[a]	5.72 ± 0.54[a]	6.49 ± 0.09[a]	6.27 ± 0.09[a]	21.43 ± 0.21
**9**	6.33 ± 0.29[a]	6.05 ± 0.63[a]	6.49 ± 0.09[a]	6.27 ± 0.09[a]	21.43 ± 0.32
**10**	7.33 ± 0.29[a]	6.39 ± 0.10[a]	4.56 ± 2.00	6.27 ± 0.09[a]	33.67 ± 0.61
**11**	7.00 ± 0.29[a]	6.39 ± 0.10[a]	5.86 ± 0.93	6.27 ± 0.09[a]	29.30 ± 0.44
**12**	7.00 ± 0.29[a]	6.39 ± 0.10[a]	6.49 ± 0.09[a]	6.27 ± 0.09[a]	22.40 ± 0.20
**13**	6.66 ± 0.50[a]	5.72 ± 0.54[a]	6.26 ± 0.37	6.27 ± 0.09[a]	22.53 ± 0.12
**14**	6.66 ± 0.50[a]	5.72 ± 0.54[a]	5.45 ± 1.17	6.27 ± 0.09[a]	21.97 ± 0.15
**15**	6.66 ± 0.50[a]	6.05 ± 0.63[a]	6.49 ± 0.09[a]	6.27 ± 0.09[a]	22.70 ± 0.46
**16**	6.33 ± 0.29[a]	5.39 ± 0.10[a]	6.49 ± 0.09[a]	6.27 ± 0.09[a]	22.57 ± 0.21
**17**	6.33 ± 0.29[a]	5.39 ± 0.10[a]	6.49 ± 0.09[a]	6.27 ± 0.09[a]	22.57 ± 0.35
**18**	6.33 ± 0.29[a]	6.05 ± 0.63[a]	6.49 ± 0.09[a]	6.27 ± 0.09[a]	21.77 ± 0.25
**19**	6.66 ± 0.50[a]	6.39 ± 0.10[a]	4.47 ± 1.82	6.27 ± 0.09[a]	29.07 ± 0.51
**20**	7.00 ± 0.29[a]	6.39 ± 0.10[a]	5.80 ± 1.16	6.27 ± 0.09[a]	30.00 ± 0.50
**21**	7.00 ± 0.29[a]	6.39 ± 0.10[a]	6.49 ± 0.09[a]	6.27 ± 0.09[a]	30.07 ± 0.61
**22**	6.33 ± 0.29[a]	5.72 ± 0.54[a]	2.06 ± 0.50	6.27 ± 0.09[a]	22.37 ± 0.15
**23**	6.66 ± 0.50[a]	6.39 ± 0.10[a]	2.83 ± 1.52	6.27 ± 0.09[a]	34.77 ± 0.55
**24**	6.33 ± 0.29[a]	5.39 ± 0.10[a]	6.02 ± 0.77	6.27 ± 0.09[a]	21.10 ± 0.20
**25**	6.66 ± 0.50[a]	6.39 ± 0.10[a]	6.23 ± 0.41	6.27 ± 0.09[a]	21.47 ± 0.25
**26**	7.00 ± 0.29[a]	6.39 ± 0.10[a]	6.49 ± 0.09[a]	6.27 ± 0.09[a]	21.60 ± 0.20
**27**	6.66 ± 0.50[a]	6.05 ± 0.48[a]	6.19 ± 0.48	5.97 ± 0.43	20.53 ± 0.35
**28**	6.33 ± 0.29[a]	6.05 ± 0.48[a]	6.39 ± 0.15	6.11 ± 0.19	21.93 ± 0.12

GC, germ carrier location; VSIV, Indiana vesiculovirus; MNV, murine norovirus; MS, *Mycobacterium senegalense*; BS, *Bacillus subtilis*; GS, *Geobacillus stearothermophilus*; T, temperature; [a], complete inactivation (differences within columns resulting from variable cytotoxic effects).

Table S5. Log$_{10}$ reduction of infectivity as mean and standard deviation (SD) of exposed germ carriers with surface temperature (mean and SD) at distinct locations in a BSL-4 laboratory suite after aerosolization of 0.5%/2.44% PAA/HP.

GC	VSIV	MNV	MS	BS	GS	T in °C
**1**	6.66 ± 0.33[a]	5.33 ± 0.00[a]	6.32 ± 0.02[a]	6.08 ± 0.04[a]	6.24 ± 0.43	23.03 ± 0.74
**2**	6.66 ± 0.34[a]	6.00 ± 0.58[a]	6.32 ± 0.02[a]	6.08 ± 0.04[a]	6.63 ± 0.24[a]	22.27 ± 0.50
**3**	6.66 ± 0.34[a]	5.66 ± 0.58[a]	6.32 ± 0.02[a]	6.08 ± 0.04[a]	6.63 ± 0.24[a]	22.27 ± 0.47
**4**	6.66 ± 0.34[a]	6.00 ± 0.58[a]	6.32 ± 0.02[a]	6.08 ± 0.04[a]	6.63 ± 0.24[a]	23.70 ± 0.70
**5**	6.66 ± 0.34[a]	5.33 ± 0.00[a]	6.32 ± 0.02[a]	6.08 ± 0.04[a]	6.63 ± 0.24[a]	23.90 ± 0.17
**6**	6.66 ± 0.34[a]	6.00 ± 0.58[a]	6.32 ± 0.02[a]	6.08 ± 0.04[a]	6.63 ± 0.24[a]	23.70 ± 0.10
**7**	6.66 ± 0.34[a]	6.00 ± 0.58[a]	6.32 ± 0.02[a]	6.08 ± 0.04[a]	6.60 ± 0.19	22.67 ± 0.55
**8**	6.66 ± 0.34[a]	5.66 ± 0.58[a]	6.32 ± 0.02[a]	6.08 ± 0.04[a]	6.07 ± 0.73	23.93 ± 0.29
**9**	6.66 ± 0.34[a]	6.33 ± 0.00[a]	6.32 ± 0.02[a]	5.34 ± 1.26	6.63 ± 0.24[a]	26.63 ± 1.46
**10**	6.66 ± 0.34[a]	6.00 ± 0.58[a]	6.32 ± 0.02[a]	6.08 ± 0.04[a]	6.63 ± 0.24[a]	25.80 ± 0.00
**11**	6.66 ± 0.34[a]	6.00 ± 0.58[a]	6.06 ± 0.45	6.08 ± 0.04[a]	6.44 ± 0.09	25.77 ± 0.21
**12**	6.66 ± 0.34[a]	6.00 ± 0.58[a]	6.32 ± 0.02[a]	5.72 ± 0.60	6.63 ± 0.24[a]	26.13 ± 0.35
**13**	6.66 ± 0.34[a]	5.66 ± 0.58[a]	6.32 ± 0.02[a]	6.08 ± 0.04[a]	6.63 ± 0.24[a]	22.97 ± 0.15
**14**	6.66 ± 0.34[a]	6.33 ± 0.00[a]	6.32 ± 0.02[a]	5.80 ± 0.46	6.63 ± 0.24[a]	33.00 ± 0.70
**15**	6.66 ± 0.34[a]	5.33 ± 1.00	6.00 ± 0.55	1.65 ± 0.22	4.48 ± 0.23	52.40 ± 3.34
**16**	6.66 ± 0.34[a]	6.00 ± 0.58[a]	6.32 ± 0.02[a]	6.08 ± 0.04[a]	5.99 ± 1.23	23.80 ± 0.56
**17**	6.66 ± 0.34[a]	5.66 ± 0.58[a]	6.22 ± 0.17	5.05 ± 1.77	6.63 ± 0.24[a]	22.13 ± 1.33
**18**	6.66 ± 0.34[a]	6.00 ± 0.58[a]	6.32 ± 0.02[a]	5.72 ± 0.60	6.63 ± 0.24[a]	24.53 ± 0.55
**19**	6.66 ± 0.34[a]	5.66 ± 0.58[a]	6.32 ± 0.02[a]	6.08 ± 0.04[a]	6.63 ± 0.24[a]	21.30 ± 0.36
**20**	6.66 ± 0.34[a]	5.66 ± 0.58[a]	6.09 ± 0.40	4.98 ± 1.88	6.63 ± 0.24[a]	33.40 ± 0.53
**21**	6.66 ± 0.34[a]	5.66 ± 0.58[a]	6.32 ± 0.02[a]	6.08 ± 0.04[a]	6.63 ± 0.24[a]	22.70 ± 0.36
**22**	6.66 ± 0.34[a]	5.66 ± 0.58[a]	6.32 ± 0.02[a]	6.08 ± 0.04[a]	6.63 ± 0.24[a]	22.63 ± 0.21
**23**	6.66 ± 0.34[a]	5.66 ± 0.58[a]	5.51 ± 1.43	6.08 ± 0.04[a]	6.63 ± 0.24[a]	22.10 ± 0.36
**24**	6.66 ± 0.34[a]	6.00 ± 0.58[a]	6.32 ± 0.02[a]	6.08 ± 0.04[a]	6.63 ± 0.24[a]	24.33 ± 0.42
**25**	6.66 ± 0.34[a]	5.66 ± 0.58[a]	6.32 ± 0.02[a]	6.08 ± 0.04[a]	6.63 ± 0.24[a]	22.47 ± 0.42
**26**	6.66 ± 0.34[a]	5.66 ± 0.58[a]	6.32 ± 0.02[a]	6.08 ± 0.04[a]	6.63 ± 0.24[a]	20.23 ± 0.75
**27**	6.66 ± 0.34[a]	6.00 ± 0.58[a]	5.83 ± 0.85	6.08 ± 0.04[a]	6.63 ± 0.24[a]	24.67 ± 1.21
**28**	6.66 ± 0.34[a]	5.33 ± 0.00[a]	6.32 ± 0.02[a]	6.08 ± 0.04[a]	6.63 ± 0.24[a]	20.60 ± 0.20
**29**	6.66 ± 0.34[a]	5.66 ± 0.58[a]	6.32 ± 0.02[a]	6.08 ± 0.04[a]	6.63 ± 0.24[a]	22.07 ± 0.25
**30**	6.66 ± 0.34[a]	6.00 ± 0.58[a]	6.32 ± 0.02[a]	5.58 ± 0.91	6.63 ± 0.24[a]	22.53 ± 0.25
**31**	6.66 ± 0.34[a]	5.66 ± 0.58[a]	6.32 ± 0.02[a]	6.08 ± 0.04[a]	6.63 ± 0.24[a]	22.33 ± 0.15
**32**	6.66 ± 0.34[a]	6.00 ± 0.58[a]	6.32 ± 0.02[a]	6.08 ± 0.04[a]	6.63 ± 0.24[a]	22.57 ± 0.31

GC, germ carrier location; VSIV, Indiana vesiculovirus; MNV, murine norovirus; MS, *Mycobacterium senegalense*; BS, *Bacillus subtilis*; GS, *Geobacillus stearothermophilus*; T, temperature; [a], complete inactivation (differences within columns resulting from variable cytotoxic effects).
